# Enhanced Herbicide Metabolism and Target-Site Mutations Confer Multiple Resistance to Fomesafen and Nicosulfuron in *Amaranthus retroflexus* L.

**DOI:** 10.3390/biology12040592

**Published:** 2023-04-13

**Authors:** Cheng Yang, Hao Wang, Yunxia Duan, Feng Bei, Sisi Jia, Jinxin Wang, Hengzhi Wang, Weitang Liu

**Affiliations:** 1 College of Plant Protection, Shandong Agricultural University, Tai’an 271018, China; 2 College of Plant Protection, Nanjing Agricultural University, Nanjing 210095, China; 3 Tai’an Customs, Tai’an 271000, China

**Keywords:** P450s, GSTs, broad-spectrum resistance, metabolic mechanisms, *Amaranthus retroflexus* L.

## Abstract

**Simple Summary:**

*Amaranthus retroflexus* L. is the most prevalent broadleaf weed in autumn crops such as soybean and corn in northeastern China. In this study, we investigated the target-site resistance mechanism of HW-01 and ST-1 populations of *A. retroflexus*, which are resistant to fomesafen and nicosulfuron, and the non-target-site resistance of cytochrome P450s- and GSTs-based herbicide metabolic was also confirmed in the HW-01 population. This study helps to provide scientific and theoretical guidance for the control of resistant populations of *A. retroflexus.*

**Abstract:**

*Amaranthus retroflexus* L. is a highly competitive broadleaf weed of corn–soybean rotation in northeastern China. In recent years, the herbicide(s) resistance evolution has been threatening its effective management in crop fields. One resistant *A. retroflexus* (HW-01) population that survived the protoporphyrinogen oxidase (PPO) inhibitor fomesafen and acetolactate synthase (ALS) inhibitor nicosulfuron applied at their field-recommended rate was collected from a soybean field in Wudalianchi City, Heilongjiang Province. This study aimed to investigate the resistance mechanisms of fomesafen and nicosulfuron and determine the resistance profile of HW-01 to other herbicides. Whole plant dose–response bioassays revealed that HW-01 had evolved resistance to fomesafen (50.7-fold) and nicosulfuron (5.2-fold). Gene sequencing showed that the HW-01 population has a mutation in *PPX2* (Arg-128-Gly) and a rare mutation in *ALS* (Ala-205-Val, eight/twenty mutations/total plants). In vitro enzyme activity assays showed that ALS extracted from the HW-01 plants was less sensitive to nicosulfuron (3.2-fold) than ST-1 plants. Pre-treatment with the cytochrome P450 inhibitors malathion, piperonyl butoxide (PBO), 3-amino-1,2,4-triazole (amitrole), and the GSTs inhibitor 4-chloro-7-nitrobenzofurazan (NBD-Cl) significantly increased fomesafen and nicosulfuron sensitivity in the HW-01 population compared with that of the sensitive (S) population ST-1. Moreover, the rapid fomesafen and nicosulfuron metabolism in the HW-01 plants was also confirmed via HPLC-MS/MS analysis. Furthermore, the HW-01 population showed multiple resistance (MR) to PPO, ALS, and PSII inhibitors, with resistance index (*RI*) values ranging from 3.8 to 9.6. This study confirmed MR to PPO-, ALS-, and PSII-inhibiting herbicides in the *A. retroflexus* population HW-01, as well as confirming that the cytochrome P450- and GST-based herbicide metabolic along with TSR mechanisms contribute to their multiple resistance to fomesafen and nicosulfuron.

## 1. Introduction

*Amaranthus retroflexus* L. is a widely distributed and troublesome annual broadleaf weed in northern China [1,2]. It is a monoecious species that is highly self-fertilized with occasional outcrossing, and due to large-scale seed production (about 1.5 million seeds), *A. retroflexus* exhibits strong adaptability [3]. More importantly, from emergence through to harvest, *A. retroflexus*, as a C4 plant, is constantly competing with the crop for nutrients, water, and light. These factors result in the severe production loss of soybean (losses up to 58%), corn (losses of between 5–34%), and other crops [4,5,6,7].

Protoporphyrinogen oxidase (PPO) is the last common enzyme that produces heme and chlorophyll in tetrapyrrole biosynthesis [8]. Inhibition of PPO enzymes with herbicides leads to the accumulation of protogen IX substrates of these enzymes. Protogen IX overflows in the cytoplasm under normal environmental conditions and is oxidized by catalase to proto IX, which, in turn, is oxidized to singlet oxygen in sunlight [9]. Ultimately, chlorophyll and carotene are lost, and membrane leakage occurs, drying up the cells and organelles and rapidly disintegrating them, resulting in plant death [10,11]. PPO-inhibitors, such as fomesafen and fluoroglycofen-ethyl, were predominantly used to control *A. retroflexus* and other grass weeds in soybean fields in China. In addition, ALS- inhibitors have been widely used in corn and soybean fields due to their ability to control many weed species, their low toxicity to mammals, and high selectivity in the world of major crops. Therefore, sulfonylureas herbicides, including nicosulfuron, thifensulfuron-methyl, and halosulfuron-methyl have been popularized and used successively in corn fields in China. Since the 1980s, Nicosulfuron has been the most popular herbicide used in corn fields to control weeds [12]. However, the repeated and extensive use of these herbicides (especially in the corn and soybean rotation regions) has led to the evolution of resistance to PPO and ALS inhibitors [1,2]. As of now, 14 weed species have evolved resistance to PPO inhibitors and 170 weed species have evolved resistance to ALS inhibitors worldwide [13].

For the target-site resistance (TSR) mechanism involved in PPO inhibitors-resistant weed species, the deletion of glycine residues at site 210 of the PPO enzyme was confirmed to be responsible for the resistance of PPO inhibitors in *A. tuberculatus* and *A. palmeri* [14,15]. In addition, point mutations in the *PPX2* gene (Arg-128-Leu, Arg-128-Gly/Met, and Gly-399-Ala) and *PPX1* gene (Ala-212-Thr) have also been reported to confer resistance to PPO inhibitors in several other species including *Ambrosia artemisiifolia* L., *A. palmeri*, and *A. tuberculatus* [16,17,18,19,20]. However, compared with the *PPX* gene, the *ALS* gene is more prone to resistance mutations, with twenty-nine different amino acid substitution mutations found in eight sites of one hundred sixty-nine species of weeds (Ala-122, Pro-197, Ala-205, Asp-376, Arg-377, Trp-574, Ser-653, and Gly-654) [21]. In previous studies, the resistance mechanism of *A. retroflexus* to PPO and ALS inhibitors was mainly focused on TSR, but the non-target-site resistance (NTSR) was still not well known [2,22,23].

NTSR is another mechanism in weeds that confers resistance to herbicides [24]. The increased activity of cytochrome P450 monooxygenases (P450s), glutathione-S-transferases (GSTs), or other enzyme systems in some herbicide-resistant populations plays an important role in herbicide metabolism [25]. In contrast to TSR, NTSR confers a greater resistance level to multiple groups of herbicides [26,27]. Recently, cytochrome P450- and GST-based non-target site mechanisms were also confirmed to be responsible for fomesafen resistance in *A. palmeri* populations from Randolph County, Arkansas [28].

In the preliminary study, one suspected resistant *A. retroflexus* population (HW-01) collected from a soybean field (with corn rotation) in Heilongjiang Province was confirmed to be multiple-resistant to PPO inhibitor fomesafen and ALS inhibitor nicosulfuron via a single herbicide dose screen test. The purposes of this study were to (1) characterize the sensitivity to different PPO inhibitors, ALS inhibitors, and a PSII inhibitor in a suspected resistant *A. retroflexus* population (HW-01); (2) identify the target site-based mechanism delivering resistance to fomesafen and nicosulfuron in the HW-01 population; (3) evaluate the effect of P450 and GST inhibitors on fomesafen and nicosulfuron resistance in the HW-01 population; (4) investigate the difference in fomesafen and nicosulfuron metabolism between the HW-01 population and the susceptible population of *A. retroflexus* (ST-1).

## 2. Materials and Methods

### 2.1. Plant Materials

In September 2017, seeds of a suspected resistant *A. retroflexus* population (HW-01) were collected from a soybean field with a continuous application history of fomesafen or nicosulfuron (>10 years) in Wudalianchi City (48.51° N, 126.13° E), Heilongjiang Province. In these regions, the growers found that fomesafen had poor control of *A. retroflexus* under the field-recommended rate. An herbicide-susceptible population of *A. retroflexus* (ST-1) was collected from Mountain Tai (36.05° N, 117.03° E), Tai’an City, Shandong Province, where there is no history of herbicide application. Mature seeds of suspected resistant (R, HW-01) and susceptible (S, ST-1) *A. retroflexus* populations were randomly collected from at least 50 individual plants, and the sample size was approximately 0.2 ha. After drying, the seeds were stored at 4 °C in paper bags until use.

### 2.2. Whole-Plant Dose–Response Experiments

For accelerating germination, *A. retroflexus* seeds were placed in Petri dishes containing two layers of Whatman No. 1 filter paper and 6 mL of deionized water. Then, the Petri dish was placed in the growth chamber (33/23 °C, 12/12 h day/night) for cultivation. After the radicle germinated, 10 seedlings (after thinned) were planted in a plastic pot measuring 15 cm in diameter and 12 cm in height and containing loam. The soil organic matter content was 1.7% and filtered using a 3-mm sieve. Plants were grown in the greenhouse with a 14-h photoperiod and a temperature of 25/15 °C day/night. The pots were watered every two days to maintain moisture.

Post-emergence application was conducted when seedlings reached the 3–4-leaves stage using a mobile nozzle cabinet sprayer equipped with flat fan nozzles (TeeJet 9503EVS, Greenman Machinery, Beijing, China) delivering a spraying volume of 450 L ha^−1^ at 280 KPa. Based on the results of preliminary experiments, dose–response tests were conducted with applications of nicosulfuron and fomesafen and five other herbicides to determine the level of resistance of HW-01 and ST-1 to these herbicides, with the higher dose used for HW-01; the herbicides as well as the doses are shown in Table 1. At 21 days after treatment (DAT), the aboveground materials from each pot were harvested and oven-dried at 80 °C for 72 h in an air-blowing box (Model DHG-9140A, Changzhou Noki Instrument Co., Ltd., Changzhou, China). Then, the dry weights were recorded. The aboveground dry weight of the plants obtained here was divided by the dry weight of the untreated control, and the result was expressed as a percentage. The experiment was designed in a completely randomized design, and the whole experiment was repeated twice.

### 2.3. Gene Sequencing and In Vitro Assay of ALS Activity

The fresh leaf tissues from each population (R, HW-01; S, ST-1; 20 individuals per population) at the 3–4-leaves stage were harvested and stored at −80 °C. For *ALS*, *PPX1*, and *PPX2* gene sequencing, RNA extraction, primers, and methods were identical to our previous report [2], and the primer pairs used to amplify *ALS*, *PPX1*, and *PPX2* of *A. retroflexus* are shown in Table S1. Then, the in vitro activity of ALS was extracted and determined according to the method described by Yu et al. and Han et al. [29,30]. The nicosulfuron (97%, Shandong Rainbow chemical Co., Ltd., Weifang, China, provided) concentrations used for the in vitro activity assays were 0.02, 0.2, 2, 20, 200, 2000 µM for the HW-01 population while 0.001, 0.01, 0.1, 1, 10, 100 µM were used for the ST-1 population. Two ALS extractions were performed for each population. The activity of each ALS extract was measured in three technical replicates and averaged.

### 2.4. Effect of P450 and GST Inhibitors on Fomesafen and Nicosulfuron Resistance

To test whether metabolic resistance was involved in the fomesafen and nicosulfuron resistance found in the HW-01 population, R and S plants (3–4-leaves stage) were treated with fomesafen and nicosulfuron, respectively, with or without the following cytochrome P450s inhibitors: malathion (Binnong, Shandong, China) at 1500 g ai ha^−1^; PBO (TCI, Shanghai, China) at 1500 g ai ha^−1^; amitrole (TCI, Shanghai, China) at 13.1 g ai ha ^−1^; and GST-inhibitor NBD-Cl (TCI, Shanghai, China) at 270 g ai ha ^−1^. Malathion, PBO, and amitrole are indicators of P450-mediated metabolic resistance and NBD-Cl is an indicator of GST-mediated metabolic resistance in weeds [31]. All cytochrome P450 inhibitors were applied 2 h before the treatment with fomesafen or nicosulfuron. In contrast, NBD-Cl was applied to plants 2 d before herbicide treatment according to the procedure outlined by Varanasi et al. and Ma et al. [28,32,33]. The application rates of fomesafen and nicosulfuron are described in Table 1. These P450- and GST-inhibitory bioassays were performed simultaneously in whole-plant dose–response experiments. The methods described above were equally applicable to the plant-growing and herbicide applications. At 21 DAT, the aboveground dry weights of the plants were recorded and expressed as percentages of the control group. The whole experiment was double-repeated and had a completely random design.

### 2.5. Analysis of Fomesafen and Nicosulfuron Metabolism in A. retroflexus

Technical-grade fomesafen (98%) and nicosulfuron (97%) were provided by Shandong Binnong Technology Co., Ltd., Binzhou, China and Shandong Rainbow chemical Co., Ltd., respectively. Micropipettes were used to apply 4.0 µg of fomesafen and 1.0 µg of nicosulfuron on S and HW-01 plants at the 3–4-leaves stage (4.0 or 1.0 µg per individual), respectively. After 1, 3, 5, 7, and 9 days of herbicide treatment, plants of S and R were selected for extraction, and the method was based on the one described by Bai et al. [34], with five time samples and three replicates. The experiment was created in a completely randomized design, and the whole experiment was carried out twice. The HPLC–MS/MS parameters are listed in Table 2, and validation of the analysis methods is in Supplementary Table S2.

Instrumentation (Thermo Fisher, Vanquish UHPLC-TSQ Quantis, Waltham, MA, USA) and HPLC–MS/MS analytical conditions of fomesafen were for separation using a C_18_ column (Agilent Eclipse Plus C_18_) at a flow rate of 0.3 mL min^−1^ at 30 °C. Mobile phase A was 0.1% formic acid in the water, while mobile phase B was methanol (LC grade). The injected sample was subjected to gradient elution at 90% component A (10%B), and the B component reached 92% (8%A) in 2.5 min, followed by adjustment of the mobile phase ratio to 90%A and 10%B in 3.5 min, which was maintained in this state for 1.5 min, after which it returned to the initial state. Detection conditions were conducted with positive ions (3500V), negative ions (2800V), sheath gas (30 Arb), aux gas (5 Arb), sweep gas (0 Arb), ion transfer tube temp (325 °C), vaporizer (350 °C). The HPLC-MS/MS parameters include min dwell time (ms) 124.00, RF lens (V) 204.

Nicosulfuron was conducted using an Acquity UPLC™ system (Waters, Milford, CT, USA) and separated using BEH C_18_ column (Waters). The gradient elution (0.4 mL min^−1^) started with 10% component A (mobile phase A: water; mobile phase B: methanol; LC grade) at the time of sample injection and was maintained for 6 min; then, it linearly increased to 90%A (10%B) at 8 min, returning to the initial state at 10 min. The MS/MS analysis conditions were performed with a desolvation temperature of 400 °C, source temperature of 110 °C, capillary voltage of 3.20 kV, desolvation gas (N_2_) flow of 520 L h^−1^, and cone gas (N_2_) flow of 82 L h^−1^. In addition, multiple reaction monitoring (MRM) and a cone voltage of 45 V were utilized. Standard sample mass spectrograms of fomesafen and nicosulfuron were listed in Figure S1.

### 2.6. Statistical Analyses

Except for gene sequencing experiments, all data from twice-repeated experiments were analyzed via ANOVA (SPSS v19.0, IBM, Armonk, NY, USA). The data generated from two runs were pooled, as the test for homogeneity of variance showed that the variance across runs was similar.

SigmaPlot (Version12.5; SigmaPlot Software Inc., San Jose, CA, USA) was used for further analysis through the following equation:

*y* = *c* + (*d* − *c*)/{1 + exp [*b* (log*x* − log*ED*_50_)]}

where *b* is the relative slope around the herbicide dose resulting in 50% growth inhibition or 50% ALS activity inhibition, *c* is the lower limit, and *d* is the upper limit. In the regression equation, the independent variable (*x*) was the herbicide rate, the dependent variable (*y*) was the growth response (percentage of the untreated control) or ALS activity (percentage of untreated control), and the *ED*_50_ represented the *GR*_50_ (the dose causing a 50% dry weight growth reduction in the aboveground) or *I*_50_ (the dose causing 50% ALS activity inhibition). The resistance index (*RI*) was calculated by dividing the *GR*_50_ *or I*_50_ value of the resistant population by that of the susceptible population.

*GR*_50_ reduction was calculated by calculating the *GR*_50_ value for fomesafen or nicosulfuron alone minus the *GR*_50_ value for the application of fomesafen or nicosulfuron plus inhibitors divided by the *GR*_50_ value for the application of fomesafen or nicosulfuron alone, and the result was expressed as a percentage.

In metabolic assay experiments, the residual dose was residual herbicide in *A. retroflexus* measured via HPLC-MS/MS, uptake dose was calculated using the total herbicide applied in leaves minus herbicide residue in acetonitrile washed off, and metabolism dose was calculated using the uptake dose minus the residual dose.

All the data are presented as means of replicates ± standard error (SE), and the means were separated using Fisher’s protected least significant difference (LSD) test at the *p* < 0.05 significance level.

## 3. Results

### 3.1. Multiple Resistance to Fomesafen and Nicosulfuron and Target-Site Mutation(s) Identification in A. retroflexus

**Figure 1:**
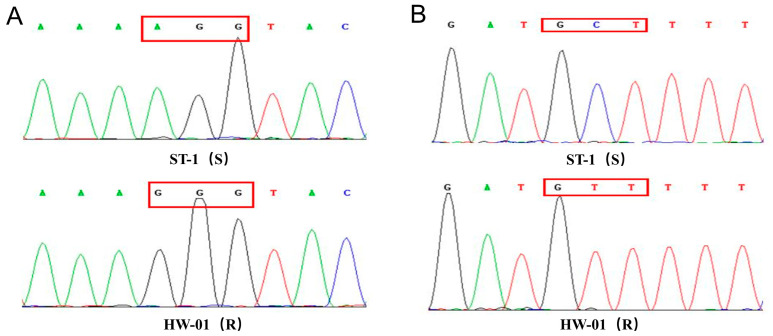
Target-site gene sequencing results indicating (**A**) the Arg-128-Gly mutation in the *PPX2* gene, (**B**) the Ala-205-Val (frequency of 40%) in the *ALS* gene in HW-01 (R), compared with ST-1(S).

Dose–response experiments confirmed that the suspected resistant population HW-01 (R) was highly resistant to fomesafen, while the ST-1 (S) population was sensitive (Table 3). The *GR*_50_ values of fomesafen to the ST-1 and HW-01 populations were 4.6 g ha ^–1^ and 235.7 g ha^–1^, respectively, which results in *RI* values (HW-01/ST-1) of 51-fold resistance. In addition, the *GR*_50_ values of nicosulfuron were 3.7 g ha^−1^ for ST-1 and 19.2 g ha^−1^ for HW-01, indicating the *RI* value was 5.2-fold and showed moderate resistance. In addition, gene sequencing revealed that all 20 detected plants of the HW-01hold Arg-128-Gly (AGG to GGG) mutation in the *PPX2* gene compared with the susceptible population (Figure 1A). However, no single nucleotide polymorphism was observed in the amplified fragments of the HW-01 population compared with the *PPX1* gene sequence of the ST-01 population. However, there was a remarkable phenomenon that a considerable percentage (12/20) of the HW-01 population had no mutation (Ala-205-Val, GCT to GTT) in the *ALS* gene and developed resistance to nicosulfuron (Figure 1B). To further explore the TSR of the HW-01 population to nicosulfuron, it is necessary to determine the activity of ALS in vitro.

### 3.2. In Vitro Assay of ALS for Nicosulfuron Activity

**Figure 2:**
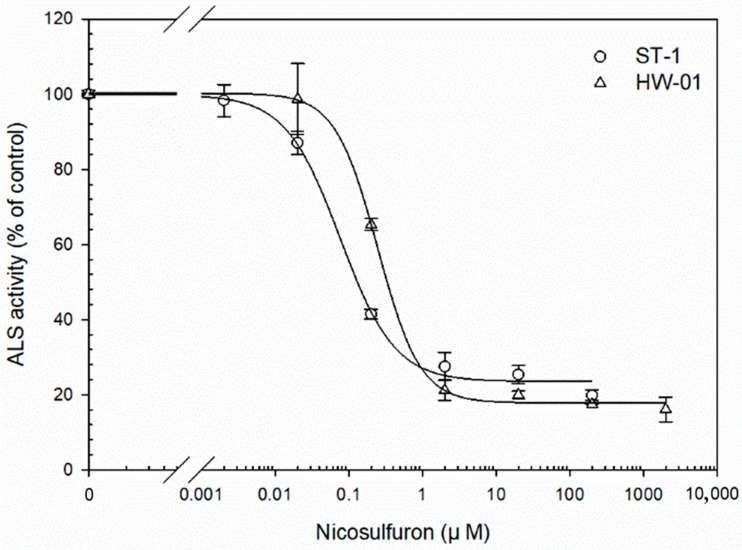
Inhibition of ALS activities in the HW-01 population (R) and ST-1 population (S) to nicosulfuron. The data represent the mean ± SE of two extractions; each treatment was repeated three times.

The results of the in vitro ALS assay showed that the total ALS activity between the HW-01 and ST-1 populations was approximately similar in the absence of nicosulfuron (15.11 ± 0.48 nmol of acetoin mg^−1^ protein min^−1^ and 16.78 ± 0.53 nmol of acetoin mg^−1^ protein min^−1^, respectively). However, the addition of nicosulfuron in the reaction almost completely inhibited ALS activity (when concentration ≥ 2 μM; Figure 2) in both S and HW-01 plants. The *I*_50_ values of HW-01 and ST-1 plants were 0.24 μM and 0.076 μM, respectively, which results in a *RI* value that is 3.2-fold lower (Figure 2).

### 3.3. Impact of P450 and GST Inhibitors on Fomesafen and Nicosulfuron Resistance

**Figure 3:**
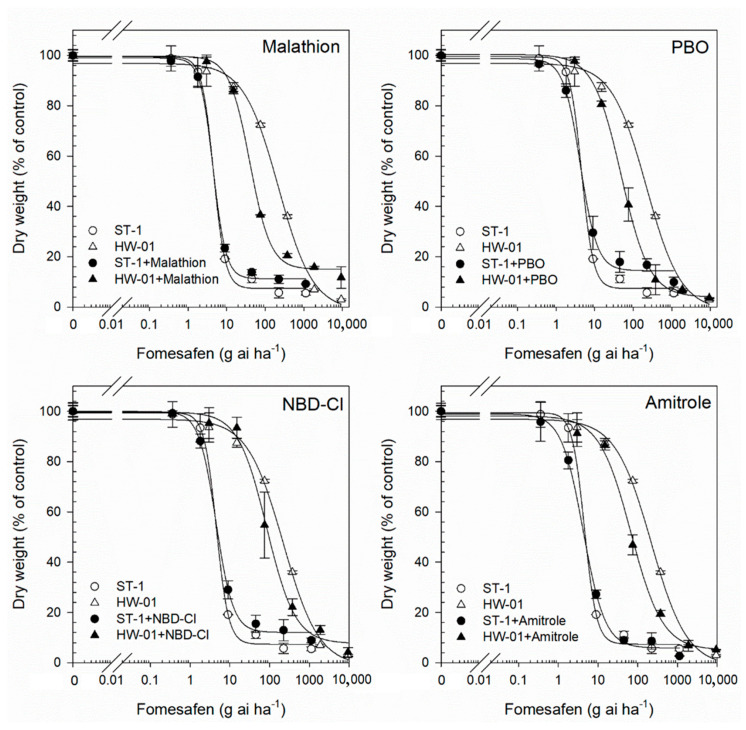
The dose–response curves of the HW-01 population (R) and ST-1 population (S) to fomesafen following cytochrome P450 inhibitors (malathion, PBO and amitrole) and GST-inhibitor (NBD-Cl) treatment.

**Figure 4:**
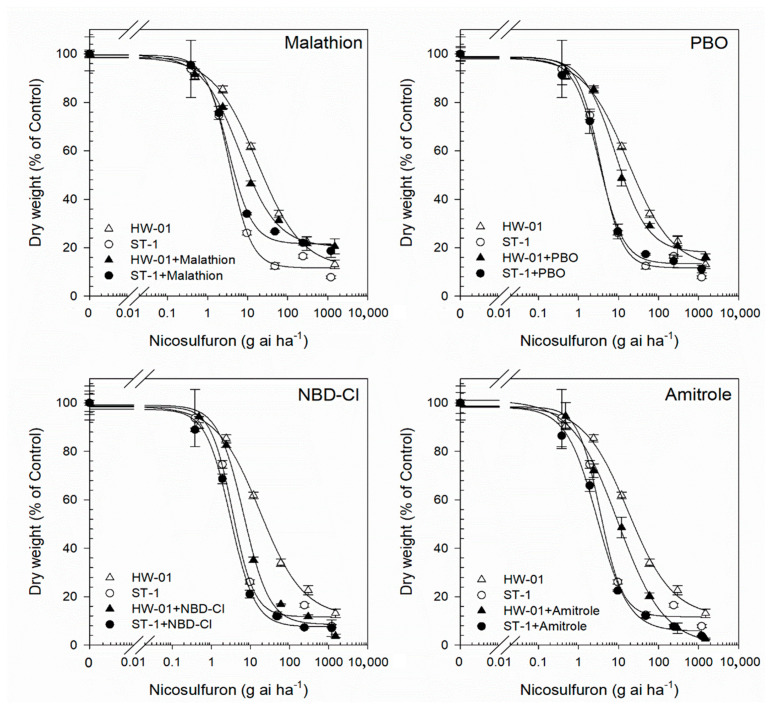
The dose–response curves of the HW-01 population (R) and ST-1 population (S) to nicosulfuron following cytochrome P450 inhibitors (malathion, PBO and amitrole) and GST-inhibitor (NBD-Cl) treatment.

The P450 and GST inhibitors used alone had no significant effects on the plant growth of either the HW-01 or ST-1 populations. However, pre-treatment with malathion, PBO, and amitrole significantly increased the toxicity of fomesafen to HW-01 plants, with *GR*_50_ values being significantly reduced by 83%, 80%, and 68%, respectively (Table 4, Figure 3). By comparison, the pre-treatment of malathion, PBO, and amitrole caused *GR*_50_ values of fomesafen to reduce by 1.5%, 5.9%, and 5.1%, respectively, in the ST-1 population (Table 4, Figure 3). For the effect of P450 and GST inhibitors on nicosulfuron sensitivity in both populations, results showed that the *GR*_50_ reductions seen in HW-01 plants caused by malathion, PBO, and amitrole plus nicosulfuron were 66%, 53%, and 60% (Table 4, Figure 4), respectively, compared to nicosulfuron alone. In contrast, no significant reductions in the *GR*_50_ values of nicosulfuron were observed in the ST-1 population with or without the pre-treatment of malathion, PBO, and amitrole (*GR*_50_ reductions were 8.6%, 3.2%, and 3.3%, respectively, Table 4, Figure 4). These results indicated that the cytochrome P450 inhibitors PBO, amitrole, and malathion had effects on the fomesafen and nicosulfuron resistance in *A. retroflexus*. Moreover, GST-inhibiting NBD-Cl pre-treatment led to fomesafen *GR*_50_ values being reduced by 75% and 3.4% (Table 4, Figure 3) for the HW-01 population and ST-1 population, respectively. Additionally, the pre-treatment of NBD-Cl led to nicosulfuron *GR*_50_ values being reduced by 64% and 5.3% (Table 4, Figure 4) for the HW-01 population and ST-1 population, respectively. Therefore, these findings suggest that the enhanced herbicide metabolism mediated by P450s and/or GSTs contribute to the fomesafen and nicosulfuron resistance in *A. retroflexus*.

### 3.4. Fomesafen and Nicosulfuron Metabolism in A. retroflexus

**Figure 5:**
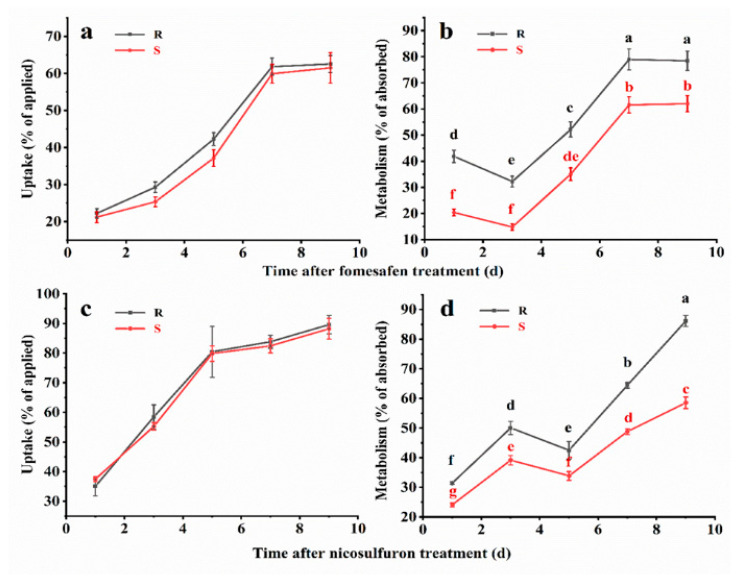
Herbicides uptake in the HW-01 population (R) and ST-1 population (S). *Amaranthus retroflexus* L. plants at 1, 3, 5, 7 and 9 d after herbicides treatment (DAT) ((**a**) Fomesafen, (**c**) Nicosulfuron). Herbicides metabolism rate in the R and S plants at 1, 3, 5, 7, and 9 DAT ((**b**) Fomesafen, (**d**) Nicosulfuron). Different letters under the same herbicide treatment indicate significant differences in metabolism at the *p* < 0.05 level according to Fisher’s protected least significant difference (LSD).

The HPLC/MS-MS results showed that fomesafen and nicosulfuron absorption were not significantly differed in the R and S plants during the experiment, and over time, fomesafen and nicosulfuron absorption increased in all the tested plants (Table 5 and Table 6). The absorption of fomesafen in the R and S plants was 22% and 21% at 1d; 29% and 25% at 3d; 42% and 38% at 5d; 62% and 60% at 7d; and 63% and 62% at 9d, respectively (Figure 5a), and there was no significant difference in the uptake of fomesafen between resistant and sensitive *A. retroflexus* plants (*p* > 0.05). However, at each sampling time, the R plants showed a significantly higher metabolic rate of fomesafen than the S plants (*p* < 0.05) (Figure 5b). The metabolism proportions of fomesafen in the R plants were significantly greater than the corresponding values in the S plants: 42%, 33%, 52%, 79% and 78% in the R plants versus 21%, 14%, 34%, 61%, 62% in the S plants at 1, 3, 5, 7, and 9 d, respectively. In addition, the uptake of nicosulfuron in HW-01 plants was not significantly (*p* > 0.05) reduced compared with ST-1 plants. The absorption of nicosulfuron in HW-01 and ST-1 plants at 1, 3, 5, 7, and 9 d were 35%, 58%, 80%, 84%, 89%, and 37%, 55%, 79%, 82%, and 88%, respectively (Figure 5c). The metabolic rates of nicosulfuron in the HW-01 plants were also confirmed to be significantly faster than those in the ST-1 plants (*p* < 0.05), which was 31%, 50%, 42%, 64%, and 86% in the HW-01 plants compared with 24%, 39%, 34%, 49%, and 59% in the ST-1 plants at 1, 3, 5, 7, 9 d, respectively (Figure 5d). These results indicated that the metabolism of fomesafen and nicosulfuron was enhanced in the HW-01 population.

### 3.5. Dose Response to Other Herbicides

This study also determined the sensitivity of *A. retroflexus* to PPO inhibitors (fluoroglycofen-ethyl, acifluorfen, lactofen, flumioxazin, and cloransulam-methyl) and a PSII inhibitor (bentazone). The ST-1 population was susceptible to all the herbicides used in the present study. Based on the *RI* values (Table 3), the HW-01 population was 9.0-fold more resistant to fluoroglycofen-ethyl relative to the ST-1 population, 7.4-fold more resistant to acifluorfen, 9.6-fold more resistant to cloransulam-methyl, and 8.4-fold more resistant to bentazone compared with ST-1. In addition, *RI* values of 3.8 and 4.0 were observed for the HW-01 population for flumioxazin and lactofen, respectively, and compared to the *RI* values of other herbicides, the HW-01 population did not develop strong resistance to these two herbicides. As a result, HW-01 plants developed resistance to all the above-tested herbicides, but the response was various.

## 4. Discussion

PPO inhibitors, especially in China, have been used for approximately 50 years since their introduction into soybean to control broadleaf weed species [35]. For the resistance evolution of PPO inhibitors in weeds worldwide, the first PPO inhibitor-resistant *A. tuberculatus* population was reported from a field with a long history of continuous soybean production and repeated selection via acifluorfen in Kansas, USA [36]. The HW-01 population tested in this study originated from soybean fields with a history of continuous use of fomesafen or nicosulfuron for over 10 years, and the 50.1-fold increase in the resistance of the resistant *A. retroflexus* population HW-01 to fomesafen is most likely due to selection pressure caused by the continuous application of this herbicide. In addition, Lamego et al. demonstrated that the continuous use of ALS inhibitors resulted in the development of herbicide resistance in *Bidens Subalternans* [37]. Additionally, Ma et al. also pointed out that the concentrated use of herbicides often leads to the selection of genes that confer herbicide resistance in weed populations [38]. Therefore, after long-term screening with fomesafen and nicosulfuron, plants with herbicide-resistant genes may gradually become the main body of the HW-01 population through reproduction, leading to the development of resistance to these two herbicides in this population.

The in vitro ALS activity assay showed that the ALS was less susceptible to nicosulfuron in the HW-01 population than in the ST-1 population. However, the altered ALS sensitivity to nicosulfuron in HW-01 was not fully consistent with the level of nicosulfuron-resistance tested in the whole-plant dose–response experiment. This phenomenon may be attributed to several factors: firstly, the samples of the in vitro ALS activity assay were randomly harvested from the population level, and the preceding target gene sequences analysis observed that HW-01 plants had a relatively low frequency of mutations in the *ALS* gene (eight/twenty, mutations/total plants); secondly, NTSR in the HW-01 population cannot be ignored and most likely played a vital role in the resistance of *A. retroflexus* to nicosulfuron and fomesafen. Therefore, here, we further investigated the NTSR mechanism in *A. retroflexus*.

The cytochrome P450 inhibitors used in this study included the organophosphate insecticide malathion, the synergistic chemical PBO, and the herbicide amitrole, which all inhibit plant P450s and are known to target different P450s enzymes [31,39]. Ma et al. and Oliveira et al. reported that malathion combined with mesotrione, tembotrione, or topramezone increased biomass reduction and herbicide efficacy in HPPD-resistant *A. tuberculatus*, which confirmed the enhanced metabolism (NTSR) mechanism in the HPPD-resistant *A. tuberculatus* [31,33]. Varanasi et al. also documented that the fomesafen resistance could be partially reversed by P450-inhibitor malathion and GST-inhibitor NBD-Cl in *A. palmeri* [28]. In this study, the P450 inhibitors (malathion, PBO, and amitrole) and GST inhibitor (NBD-Cl) drastically reversed the resistance of the HW-01 plants to fomesafen and nicosulfuron, suggesting the presence of NTSR mediated by P450s and GSTs in HW-01 which contributes to the herbicide-resistant phenotype by enhancing herbicide metabolism. To the best of our knowledge, this is the first report on NTSR to fomesafen and nicosulfuron resistance in *A. retroflexus*, and with the continued use of multiple sites of action (SOA) herbicides, likely, populations of *A. retroflexus* in this dual resistance state (TSR coexisting with NTSR) will become more numerous. Generally, NTSR confers unpredictable cross-resistance patterns, which may threaten the possibility of herbicide mixing in delaying resistance evolution [40,41]. Worse, a new herbicide MOA is not expected to occur in the near future [42]. Therefore, NTSR increases the difficulty of herbicide control, based on the emergence of TSR in weeds. From this point of view, the multiple-herbicide-resistant *A. retroflexus* may be becoming troublesome in weed management in the soybean and/or corn production regions of China.

The use of P450 or GST inhibitors (malathion, PBO, amitrole, NBD-Cl) may be part of a future solution for NTSR, and it opens an avenue of research that warrants further exploration. Studies have demonstrated that synergists can reverse herbicide resistance [43,44]. However, the main problem with P450 or GST inhibitors is that these molecules may also reduce crop selectivity and have unintended environmental effects. In this study, the use of herbicides with P450 and GST inhibitors significantly reduced *GR*_50_ values compared to herbicide application alone (*p* < 0.05), and it confirmed the existence of a metabolic resistance phenotype in *A. retroflexus*. However, how P450s and GSTs mediate herbicide metabolism and which enzyme family members are involved in metabolic resistance merits need further investigation.

There have been many cases of mutations in *ALS* and *PPX2* in Amaranthus leading to their resistance to ALS and PPO inhibitors and more and more target-site mutations have been reported [17,18,21]. However, with the discovery of the first case of amaranth with non-target resistance [45], more and more studies have confirmed that non-target resistance plays an important role in the resistance of amaranth to herbicides, among which Varanasi et al. demonstrated that the population of palmer amaranth that developed non-target resistance delivered cross-resistance to PPO inhibitors, and this population developed some degree of resistance to herbicides of multiple-herbicide mechanisms of action (including ALS inhibitors) [28]. Rangani et al. revealed that palmer amaranth developed metabolic resistance to s-metolachlor in which the *GST* gene family played an important role [46]. Küpper et al. highlighted the involvement of the P450 enzyme family in the palmer amaranth detoxification of tembotrione [47]. Obenland et al. experimentally confirmed that resistance to carfentrazone-ethyl in *A. tuberculatus* arises due to the production of NTSR within this population [48]. The resistant population in this study, HW-01, experimentally confirmed the involvement of P450s and GSTs in the detoxification metabolism of nicosulfuron and fomesafen. The presence of NTSR in HW-01 may be one of the reasons for the formation of multiple resistance patterns. It has demonstrated that using low or sublethal herbicide rates was one of the main reasons attributed to the evolution of metabolic resistance, resulting in the accumulation of metabolic genes over several generations and resistance to different types of herbicides [25,40].

The HW-01 population already had a high index of resistance to PPO, ALS, and PSII inhibitors (Table 3), and the population developed multiple resistance to herbicides belonging to these chemical groups, probably because the widespread use of these three types of herbicides in soybean–corn rotation fields contributed to the formation of NTSR in HW-01. The herbicide resistance caused by multiple target-site mutations has also been reported in *A. retroflexus* and *A. palmeri* [17,49]. Multiple resistance due to more than one target gene resistance mutation has also been reported in lots of weed species, such as *Alopecurus* Linn and *Echinochloa* Beauv [50,51,52]. However, there was no history of a large-scale use of flumioxazin in China’s soybean/corn fields [53], and the HW-01population showed low resistance to flumioxazin, so growers should be vigilant about the use of flumioxazin to prevent overuse that could lead to resistance to the herbicide in *A. retroflexus*. In previous studies, it was also reported that wild radish evolved low-level resistance to diflufenican after only four applications [54,55].

The reasons for the results of this investigation were most likely diverse. In terms of TSR, a mutation in the *PPX2* gene (Arg-128-Gly) was confirmed in the HW-01 population, which could be a reason for conferring cross-resistance to flumioxazin; *PPX1* was compared and no mutation was found; however, whether there is an overexpression of *PPX1* in the HW-01 population causing TSR still needs further experimental verification. Additionally, we have demonstrated the presence of NTSR in the HW-01 resistant population; therefore, enhanced cytochrome P450- and GST-mediated herbicide metabolism may be another reason for HW-01 resistance to flumioxazin. However, NTSR has different metabolic effects on different herbicides, so whether NTSR is involved in the metabolic detoxification of HW-01 to flumioxazin and to what extent it plays a role still needs further experimental confirmation.

## 5. Conclusions

In summary, in this study, we identified one *A. retroflexus* population, which has evolved multiple resistance to PPO, ALS, and PSII inhibitors. The multiple resistance to fomesafen (PPO inhibitor) and nicosulfuron (ALS inhibitor) was endowed by P450- and GST-mediated enhanced herbicide metabolism and target-site mutations. For resistance development, the specific herbicide application history in the soybean and corn rotation of agricultural practice likely facilitated the rapid evolution of ALS and PPO inhibitor resistance in *A. retroflexus* [56]. This phenomenon poses a threat to the chemical control of weeds in crop fields, and the “co-evolution” of the target gene and metabolism for herbicide resistance should be paid attention to and further understood.

## Figures and Tables

**Table 1:** Herbicide rates applied in the whole-plant experiment.

Herbicide ^a^	Group ^b^	Application Rate (g ai ha^−1^)	
		ST-1 (S)	HW-01 (R)
Fomesafen	E	0.36, 1.8, 9, 45, 225, 1125	3, 15, 75, 375, 1875, 9375
Fluoroglycofen-ethyl	E	0.096, 0.48, 2.4, 12, 60, 300	0.72, 3.6, 18, 90, 450, 2250
Acifluorfen	E	0.58, 2.89, 14.4, 71.9, 359.5, 1797.6	3.9, 19.3, 96.3, 481.5, 2407.5, 12,037.5
Lactofen	E	0.72, 3.6, 18, 90, 450, 2250	1.01, 5.04, 25.2, 126, 630, 3150
Flumioxazin	E	0.18, 0.9, 4.5, 22.5, 112.5, 562.5	0.24, 1.2, 6, 30, 150, 750
Bentazone	C3	5.9, 29.9, 149.6, 748, 3740, 18,700	11.9, 59.9, 299.5, 1497.6, 7488, 37,440
Nicosulfuron	B	0.384, 1.92, 9.6, 48, 240, 1200	0.48, 2.4, 12, 60, 300, 1500
Cloransulam-methyl	B	0.04, 0.20, 1.0, 5.0, 25.0, 125.0	0.2, 1.0, 5.0, 25.2, 126, 630

^a^ Fomesafen (250 g/L AS, BrightMart CropScience, Foshan, China), Fluoroglycofen-ethyl (10% ME, Huifeng Biological Agriculture, Yancheng, China), Acifluorfen (21.4% AS, Hanshen Biotechnology, Qindao, China), Flumioxazin (50% WP, Sumitomo Chemical Corp, Tokyo, Japan), Bentazone (480 g/L AS, BASF SE, Ludwigshafen, Germany), Lactofen (240 g/L EC, Binnong Technology, Binzhou, China), Nicosulfuron (40 g/L OF, Zhongshan Chemical Group, Huzhou, China), Cloransulam-methyl (84% WG, Dow AgroSciences, Beijing, China). ^b^ Abbreviations: B, Inhibition of Acetolactate; C3, Inhibition of photosynthesis at PS ll—D1 Histidine 215 binders; E, Inhibition of PPO.

**Table 2:** HPLC-MS/MS conditions for fomesafen and nicosulfuron.

Herbicide	Retention Time (min)	Quantitative Ions	Qualitative Ion	Collision Energy (eV)	Scan Mode
Fomesafen	2.52	437.05/194.50	437.05/222.00	36.26 ^a^/31.45	ESI^−^
Nicosulfuron	7.76	411.06/182.17	411.06/213.18	17 ^a^/15	ESI^+^

^a^ Collision energy of the quantitative ions.

**Table 3:** Parameter values of the four-parameter log-logistic equation to calculate the *GR*_50_ values of the susceptible (ST-1) and resistant (HW-01) populations of *A. retroflexus* with the use of the whole-plant dose–response experiments. Standard errors (SE) are in parentheses.

Herbicide	Group ^a^	Populations ^b^	Regression Parameters ^c^				*GR*_50_	*RI* ^d^
			*c*	*d*	*b*	*r* ^2^		
Fomesafen	E	R	0.18 (0.005)	93.13 (0.004)	−1.10 (0.12)	0.9989	235.71 (24.46)	50.7
		S	7.37 (1.83)	98.90 (3.21)	−2.89 (0.55)	0.9980	4.65 (0.67)	
Fluoroglycofen-ethyl	E	R	4.69 (1.56)	96.08 (5.52)	−0.75 (0.20)	0.9949	90.84 (3.90)	9.0
		S	6.47 (1.07)	98.66 (4.36)	−0.83 (0.16)	0.9970	10.10 (2.27)	
Acifluorfen	E	R	6.15 (2.59)	98.40 (6.27)	−0.81 (0.23)	0.9936	447.67 (53.39)	7.4
		S	7.53 (1.96)	97.47 (3.96)	−0.81 (0.42)	0.9975	60.39 (13.09)	
Lactofen	E	R	9.66 (0.26)	93.87 (0.29)	−1.28 (0.02)	1.0000	42.61 (0.59)	4.0
		S	4.32 (1.46)	96.03 (2.74)	−1.62 (0.19)	0.9988	10.78 (0.99)	
Flumioxazin	E	R	9.99 (1.50)	96.15 (2.62)	−3.13 (0.54)	0.9985	2.28 (0.29)	3.8
		S	16.28 (2.41)	84.09 (4.19)	−2.84 (1.77)	0.9934	0.60 (0.17)	
Nicosulfuron	B	R	13.52 (3.43)	94.46 (4.18)	−0.90 (0.17)	0.9922	19.21 (3.91)	5.2
		S	11.51 (2.32)	96.38 (4.91)	−1.57 (0.29)	0.9896	3.69 (0.61)	
Cloransulam-methyl	B	R	1.12 (0.16)	95.23 (1.52)	−1.00 (0.09)	0.9992	30.57 (2.96)	9.6
		S	0.83 (2.56)	96.41 (2.07)	−0.87 (0.08)	0.9992	3.20 (0.33)	
Bentazone	C3	R	9.40 (3.72)	101.49 (5.33)	−0.83 (0.15)	0.9974	381.26 (78.48)	8.4
		S	11.22 (3.75)	112.93 (13.97)	−0.96 (0.27)	0.9936	45.52 (17.65)	

^a^ Abbreviations: B, Inhibition of Acetolactate; C3, Inhibition of photosynthesis at PS ll—D1 Histidine 215 binders; E, Inhibition of PPO. ^b^ R, resistant population HW-01; S, susceptible population ST-1. ^c^ *y* = *c* + (*d* − *c*)/{1 *+* exp [*b* (log*x* − log*ED*_50_)]}, where *b* is the relative slope around the herbicide dose resulting in 50% growth inhibition, *c* is the lower limit, *d* is the upper limit, *x* is the herbicide dose, and *y* is the growth response (percentage of the untreated control); *GR*_50_, dose required to reduce plant dry weight by 50%. ^d^ *RI* = *GR*_50_ (HW-01)/*GR*_50_ (ST-1).

**Table 4:** Effect of P450 and GST inhibitors on the resistance of fomesafen and nicosulfuron in *A. retroflexus*.

Herbicide	HW-01			ST-1		
	*GR*_50_	*GR*_50_ Reduction (%)	*p*-Value	*GR*_50_	*GR*_50_ Reduction (%)	*p*-Value
Fomesafen	235.71 (24.46)	--	--	4.65 (0.67)	--	--
Fomesafen + Malathion	40.51 (6.15) *	82.83%	0.00	4.58 (0.46)	1.51%	0.99
Fomesafen +PBO	47.91 (3.20) *	79.67%	0.00	4.39 (0.76)	5.59%	0.81
Fomesafen + Amitrole	71.98 (13.08) *	69.46%	0.00	4.41 (0.54)	5.16%	0.82
Fomesafen + NBD-Cl	85.12 (19.54) *	63.89%	0.00	4.49 (0.53)	3.44%	0.93
Nicosulfuron	19.21 (3.91)	--	--	3.69 (0.61)	--	
Nicosulfuron + Malathion	6.61 (1.42) *	65.59%	0.00	3.37 (0.58)	8.67%	0.65
Nicosulfuron + PBO	9.04 (2.00) *	52.94%	0.00	3.57 (0.42)	3.25%	0.96
Nicosulfuron + Amitrole	7.76 (2.38) *	59.60%	0.00	3.56 (0.58)	3.52%	0.95
Nicosulfuron + NBD-Cl	6.73 (1.68) *	64.97%	0.00	3.49 (0.33)	5.42%	0.86

* Within the same population, the difference in *GR*_50_ values between the use of an inhibitor plus fomesafen or niclosulfuron and fomesafen or niclosulfuron alone was significant (*p* < 0.05).

**Table 5:** Analysis of metabolic dynamics of fomesafen in *A. retroflexus* plants.

DAT ^a^	Residual Dose of Fomesafen (μg)			Uptake Dose of Fomesafen (μg)			Metabolism Dose of Fomesafen (μg)		
	HW-01	ST-1	*p*-Value	HW-01	ST-1	*p*-Value	HW-01	ST-1	*p*-Value
1	2.60 (1.40)	3.39 (0.11)	0.22	4.47 (1.11)	4.26 (1.27)	0.94	1.88 (0.23)	0.88 (0.14) *	0.00
3	3.96 (1.22)	4.34 (1.13)	0.75	5.89 (1.23)	5.07 (1.15)	0.17	1.94 (0.47)	0.73 (0.17) *	0.00
5	4.08 (1.63)	4.95 (1.29)	0.23	8.48 (1.49)	7.54 (1.94)	0.28	4.40 (1.44)	2.59 (0.40) *	0.00
7	2.67 (1.08)	4.61 (1.53) *	0.00	12.41 (1.98)	11.96 (2.21)	0.87	9.74 (1.87)	7.35 (1.00) *	0.00
9	2.75 (1.07)	4.65 (1.54) *	0.00	12.56 (2.05)	12.33 (3.54)	0.99	9.81 (1.60)	7.68 (1.28) *	0.00

^a^ DAT: days after treatment. * ST-01 was significantly different from HW-1 at the residual dose, absorbed dose, and metabolic dose (*p* < 0.05) using Fisher’s protected least significant difference (LSD) test.

**Table 6:** Analysis of metabolic dynamics of nicosulfuron in *A. retroflexus* plants.

DAT ^a^	Residual Dose of Nicosulfuron (μg)			Uptake Dose of Nicosulfuron (μg)			Metabolism Dose of Nicosulfuron (μg)		
	HW-01	ST-1	*p*-Value	HW-01	ST-1	*p*-Value	HW-01	ST-1	*p*-Value
1	1.20 (0.23)	1.42 (0.37)	0.73	1.75 (0.32)	1.87 (0.08)	0.42	0.55 (0.04)	0.45 (0.06) *	0.01
3	1.46 (0.27)	1.68 (0.18)	0.14	2.92 (0.41)	2.76 (0.13)	0.40	1.46 (0.21)	1.08 (0.11) *	0.00
5	2.32 (0.53)	2.64 (0.42)	0.30	4.02 (0.86)	3.99 (0.26)	0.93	1.70 (0.36)	1.35 (0.17)	0.07
7	1.49 (0.16)	2.11 (0.08) *	0.00	4.19 (0.22)	4.12 (0.24)	0.63	2.70 (0.09)	2.01 (0.09) *	0.00
9	0.62 (0.09)	1.83 (0.15) *	0.00	4.48 (0.31)	4.41 (0.35)	0.73	3.86 (0.12)	2.58 (0.20) *	0.00

^a^ DAT: days after treatment. * ST-01 was significantly different from HW-1 at the residual dose, absorbed dose, and metabolic dose (*p* < 0.05) using Fisher’s protected least significant difference (LSD) test.

## Data Availability

The data presented in this study are available upon request from the corresponding author.

## Supplementary Materials

The following supporting information can be downloaded at: https://www.mdpi.com/article/10.3390/biology12040592/s1. Figure S1: Typical chromatograms of fomesafen and nicosulfuro—standard and extracted *Amaranthus retroflexus* L samples [HW-01 population (R) and ST-1 population (S)]. Table S1: Primers used to amplify the *PPX1, PPX2,* and *ALS* genes of *A. retroflexus*. Table S2: The analysis method of fomesafen and nicosulfuron in *A. retroflexus* using HPLC-MS/MS was validated in terms of linearity, limit of quantification (LOQ), accuracy, and precision.
