# Supplementary material for: Enhanced Herbicide Metabolism and Target-Site Mutations Confer Multiple Resistance to Fomesafen and Nicosulfuron in Amaranthus retroflexus L
Source: Biology (Basel). 2023 Apr 13;12(4):592. doi: 10.3390/biology12040592 (PMC10135446; doi:10.3390/biology12040592)
Supplement: Supplementary file 1 [file biology-12-00592-s001.zip › biology-2233659-supplementary.pdf]

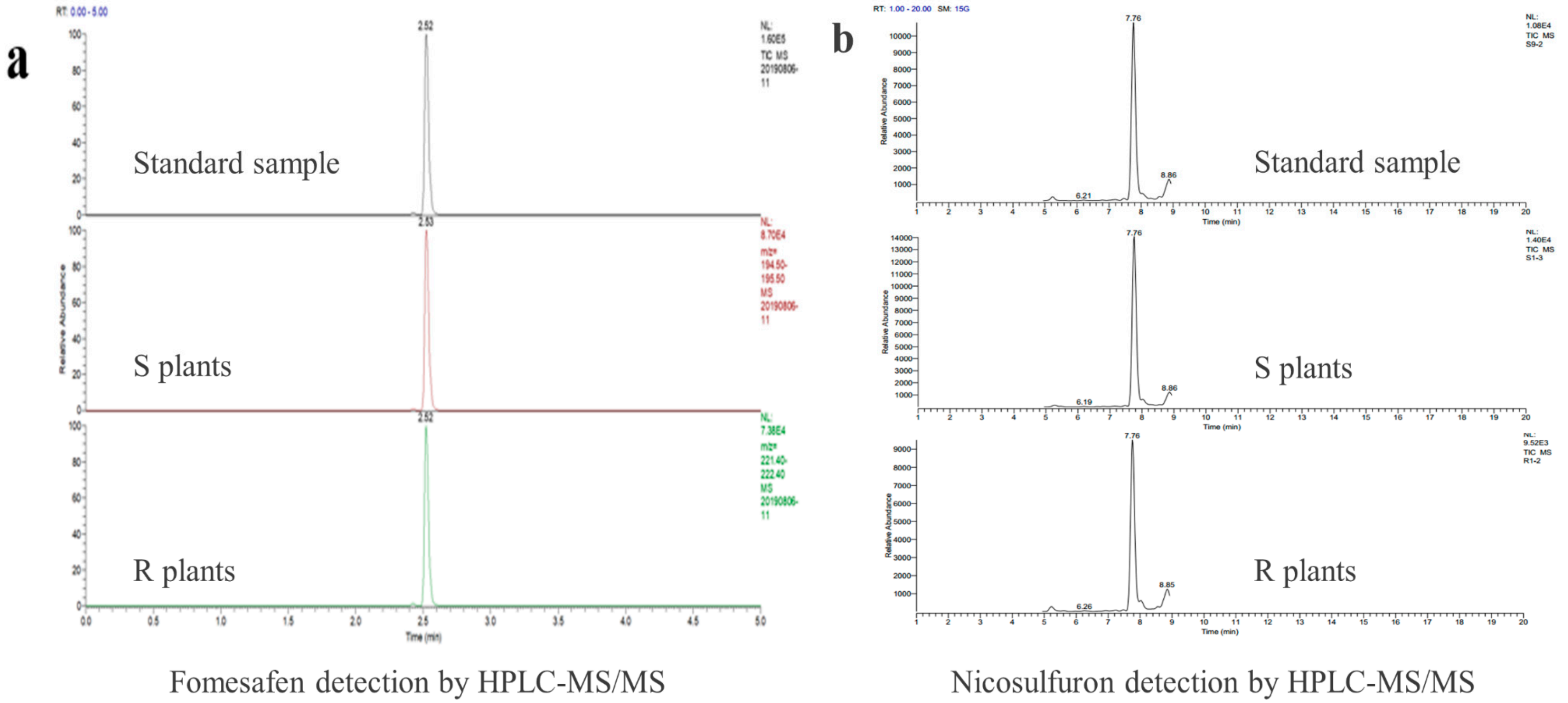

Figure S1. Typical chromatograms of fomesafen and nicosulfuron standard, and extracted *Amaranthus retroflexus* L samples [HW-01 population (R), and ST-1 population (S)].

Table S1. Primers used to amplify the *PPX1*, *PPX2*, and *ALS* gene of *A. retroflexus*.

**Primers**	**Sequence(5'-3')**	**Tm (°C)**	**Amplicon size (bp)**
PPX1			
X1-F	GAGAGAGTGCGAGAGA-GATGAG	55	1490

X1-R	CAAGATGCTG-GAGCCCTATTGAC		
PPX2			
X2-F	GCCATCGCCATTGTCAG-TTTCA	56	1450
X2-R	ATGGATGAGAAGACCGCG-TAA		
ALS			
ALS-F	TGCGATGTTCTCGTT-GAAGC	58	1711
ALS-R	CCCTTCTTCCATCAC CCT		

Table S2. The analysis method of fomesafen and nicosulfuron in *A. retroflexus* using HPLC-MS/MS was validated in terms of linearity, limit of quantification (LOQ), accuracy and precision.

Herbicide	Calibration curve	R^2	LOQ [a] (mg kg^{-1})	Fortified concentration (mg kg^{-1})	Recoveries [b] (%)
Fomesafen	y=373.09x+1941.6	0.9999	0.005	0.005	82.26±2.96
				0.05	88.51±6.23
				0.1	90.87±5.44
Nicosulfuron	y=17955.6x+7213.62	0.9995	0.005	0.005	85.60±3.74
				0.05	89.46±5.61
				0.1	92.35±6.17

[a] The LOQ was considered the lowest fortified concentration that can be quantified with acceptable accuracy and precision.

[b] Average recoveries (n=5) ± relative standard deviations (RSDs) of fomesafen and nicosulfuron at three fortified concentrations were used to assess accuracy and precision, respectively.
